# Case Report: Spontaneous Extensor Tendon Rupture of the Middle and Ring Fingers in a Patient With Multiple Autoimmune Syndrome

**DOI:** 10.1155/cro/4977682

**Published:** 2026-11-06

**Authors:** Chuancheng Huang, Baoguo Cheng, Deshuai Tian, Bin Wang, Dawei Wang, Chen Xie, Zongyu Li

**Affiliations:** ^1^ Hand Surgery Department The 960th Hospital of PLA Jinan Shandong China

**Keywords:** case report, multiple autoimmune syndrome, tendon injuries

## Abstract

Spontaneous tendon rupture is a classic complication of rheumatoid arthritis (RA), predominantly affecting the ulnar‐sided digits and extensor pollicis longus in middle‐aged and elderly patients. Spontaneous extensor tendon rupture in a young patient is uncommon. We report a 29‐year‐old female teacher with RA, Type 1 diabetes mellitus (T1DM) and hypothyroidism (collectively meeting criteria for multiple autoimmune syndrome (MAS) ≥ 3 coexisting autoimmune diseases) who received long‐term immunosuppressive and hormone replacement therapy. She initially presented with dorsal hand pain and masses diagnosed as tenosynovitis and developed complete extension loss of the right middle and ring fingers 2 weeks later. Surgical exploration confirmed extensor tendon rupture of the two digits and laxity of the extensor digiti minimi. Tendon grafting, end‐to‐side suture, and tenodesis restored active finger extension at the 3‐month follow‐up, with QuickDASH score improved from 78 points to 6 points; these represent favorable short‐term outcomes. Longer follow‐up is needed to assess the durability of these results. This case describes unusually early‐onset severe tendon rupture in a young RA patient with additional autoimmune and metabolic comorbidities, highlighting the potential need for enhanced monitoring and occupational protection in patients with MAS and high hand workload.

**Highlights**

• A rare case of spontaneous middle and ring finger extensor tendon rupture is reported in a young patient with triple autoimmune comorbidities, expanding the clinical description of severe tendon injury occurring in young patients with multiple co‐existing autoimmune disorders.
• The coexistence of RA, Type 1 diabetes mellitus (T1DM) and hypothyroidism (HT) may represent additive risk factors for tendon injury; however, the individual contribution and potential synergistic effect of each autoimmune or metabolic comorbidity cannot be established based solely on this single case.
• Combined tendon reconstruction surgery paired with multidisciplinary perioperative management achieves encouraging short‐term functional recovery at 3 months, supporting proactive tendon monitoring as a reasonable clinical consideration for high‐occupational‐hand‐load patients with multiple autoimmune syndrome (MAS).

## 1. Introduction

Spontaneous tendon rupture is a typical complication of rheumatoid arthritis (RA), usually caused by direct infiltration or erosion of tendons by RA‐induced synovitis and tenosynovitis, or by mechanical friction from osteophytes or distal ulnar dislocation [1, 2]. The extensor pollicis longus tendon [3] and Achilles tendon are commonly affected sites [4]. Middle‐aged and elderly RA patients are at higher risk of spontaneous tendon rupture [5]. MAS refers to a condition in which a single patient has three or more coexisting autoimmune diseases [6, 7]. A case of spontaneous extensor tendon rupture in a young female with RA and additional autoimmune comorbidities (T1DM and HT) is reported herein, which highlights the potential for rapid disease progression in patients with multiple coexisting autoimmune diseases and the need for multidisciplinary management.

## 2. Case Presentation

### 2.1. Patient Information

A 29‐year‐old unmarried female teacher presented to our hospital on September 5, 2025, with a 1‐week history of painful swelling on the dorsum of her right hand, accompanied by vulvar pruritus. She denied a family history of autoimmune diseases. Her medical history included T1DM diagnosed at age 10, RA diagnosed at age 18, and Hashimoto′s thyroiditis with HT diagnosed at age 26. Her long‐term medications included insulin (7 U, 3 times daily), iguratimod (25 mg, twice daily), tofacitinib citrate (11 mg, once daily), hydroxychloroquine sulfate (0.2 g, once daily) and levothyroxine sodium (50 *μ*g, once daily). She reported an average of 4 h of daily keyboard use for teaching and office work.

### 2.2. Diagnostic Assessment

#### 2.2.1. Imaging and Laboratory Findings

Initial radiographs of the right wrist showed irregular bony contours, heterogeneous bone density, and narrowed joint spaces at the scaphoid, lunate, triquetrum, pisiform, and distal radioulnar joint (DRUJ) (Figure 1A,B). Magnetic resonance imaging (MRI) confirmed triangular fibrocartilage complex injury, bone marrow edema, tenosynovitis, and joint effusion (Figure 1C,D). Vaginal secretion culture was positive for *Candida* species.

**Figure 1:**
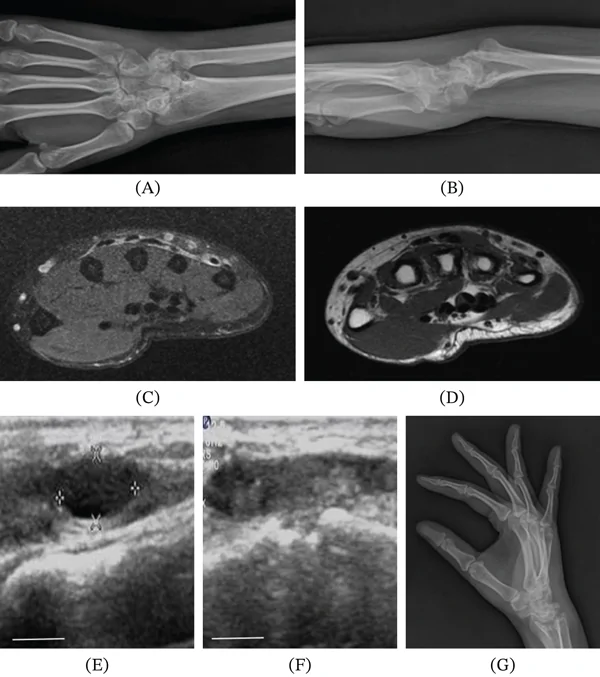
Preoperative x‐ray results ((A) anteroposterior view and (B) lateral view) show irregular bony contours, heterogeneous density, and narrowed joint spaces in the scaphoid, lunate, triquetrum, pisiform, and distal radius/ulna of the right wrist, including narrowing of the distal radioulnar joint. MRI results ((C) T1‐weighted image and (D) T2‐weighted image) reveal tenosynovitis and joint effusion; tenosynovitis is visible in both the flexor compartment (flexor carpi radialis and flexor pollicis longus tendons) and the dorsal extensor compartment, reflecting the systemic inflammatory environment. Note that the MRI was obtained before complete extensor tendon rupture was clinically apparent; the rupture was confirmed intraoperatively. Ultrasonography results ((E) transverse section and (F) longitudinal section; scale bar = 5 mm) demonstrate a cystic mass adjacent to the extensor tendons dorsal to the third and fourth metacarpal bones, accompanied by peritendinous effusion, complete tendon disruption was not identified on ultrasound. (G) One‐month postoperative follow‐up radiograph demonstrates wrist bony features comparable to preoperative images; full finger extension function was clinically achieved at the 3‐month postoperative visit.

Laboratory tests performed on September 8 revealed elevated antiglutamic acid decarboxylase antibody (1633 IU/mL, reference: 0–5), anti‐islet cell antibody (50.5 U/mL, reference: 0–7.5), thyroglobulin antibody (160 IU/mL, reference: 0–115), and thyroid peroxidase antibody (166.0 IU/mL, reference: 0–34). Thyroid‐stimulating hormone was within normal limits at 2.53 *μ*IU/mL (reference: 0.27–4.2), although free‐T4 was not measured. Glycated hemoglobin was 10.5% (reference: 3.0–6.0), indicative of poor long‐term glycemic control. Urinalysis demonstrated 2+ occult blood and elevated urinary albumin (47 mg/L, reference: 0–30). Notably, baseline erythrocyte sedimentation rate, admission C‐reactive protein (CRP), and disease activity score‐28 for RA were not documented in our medical records.

On September 23, the patient presented to the emergency department with complete loss of active extension of the right middle and ring fingers. Physical examination revealed a tender subcutaneous mass on the dorsal hand, with 0/5 motor strength of the extensor digitorum communis of the middle and ring fingers. Ultrasonography, performed by a radiologist in the emergency setting, showed a cystic mass adjacent to the extensor tendons with peritendinous effusion, but did not detect complete tendon rupture (Figure 1E,F).

#### 2.2.2. Functional Assessment (ICF Framework)

Functional assessment results were as follows:

• Body functions and structures: b730 muscle power function impairment: 0/5 strength of right middle and ring finger extensor muscles. b2801 joint mobility impairment: loss of active extension range of right middle and ring fingers. s730 structure of muscles and tendons of hand: complete discontinuity of extensor tendons of right middle and ring fingers; laxity of extensor digiti minimi tendon. s720 structure of wrist joint: erosive changes of carpal bones, narrowed joint spaces.
• Activity limitations: d440 fine hand use: inability to perform writing, typing, and pinching with the right hand. d430 lifting and carrying objects: impaired ability to grasp and lift objects with the right hand.
• Participation restrictions: d820 work and employment: inability to complete teaching tasks requiring writing and computer operation. d920 daily living: partial impairment of self‐care activities requiring right hand extension.

#### 2.2.3. Core Diagnoses With ICD‐10 Codes

Core diagnoses were as follows:

1. RA, unspecified (M06.9; limited inpatient documentation precluded more specific subcode assignment)
2. T1DM without complications (E10.9)
3. Autoimmune thyroiditis (Hashimoto′s disease) with HT (E06.3)
4. Spontaneous rupture of extensor tendons of right middle and ring fingers (M66.879)

### 2.3. Therapeutic Intervention

A multidisciplinary team completed comprehensive preoperative optimization for this complex patient. Insulin therapy was adjusted to achieve intraoperative blood‐glucose targets of 7.8–10.0 mmol/L. Baseline immunosuppressive agents included iguratimod, tofacitinib, and hydroxychloroquine; tofacitinib was withheld 3 days before surgery, whereas iguratimod and hydroxychloroquine were continued throughout the preoperative period. Preoperative vaginal candidiasis received topical treatment and resolved before surgery.

Emergency surgical exploration was performed under brachial plexus anesthesia via a dorsal longitudinal incision over the right wrist and metacarpals. Intraoperative findings included complete rupture of the extensor digitorum communis tendons of the middle and ring fingers, located at the level of the dorsal wrist over the distal radius and carpus (approximately extensor tendon Zones V–VI, within the fourth extensor compartment), with defect lengths of approximately 2.5 and 3.0 cm, respectively. The ruptured stumps were replaced by fibrous scar tissue rather than acutely lacerated ends, consistent with a chronic attritional process. No gross mechanical abrasion, fraying against a prominent ulnar head, or direct osteophyte impingement was identified during exploration; however, synovial hypertrophy and tenosynovitis were evident around the rupture sites. The extensor digiti minimi tendon was intact but significantly lax without resting tension (Figure 2A,B).

**Figure 2:**
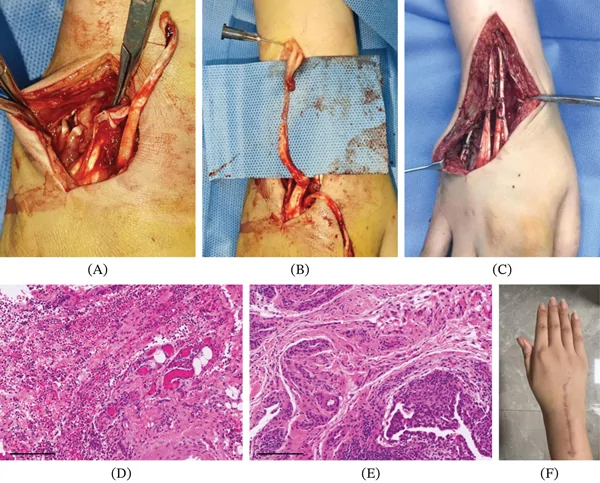
(A,B) Intraoperative exploration reveals defects in the extensor tendons of the right middle and ring fingers, replaced by fibrous scar tissue; the extensor digiti minimi tendon remained continuous but was lax and lacked tension. (C) The surgical procedure involved debridement of the degenerated tendons, reconstruction of the ring finger extensor tendon using a palmaris longus tendon graft, end‐to‐side suture of the middle finger extensor tendon stump to the ulnar side of the extensor indicis tendon, and tightening tenodesis of the extensor digiti minimi tendon. (D,E) Hematoxylin and eosin (H&E) histological staining of the paraffin‐embedded tissue sample from the tendon rupture site (20x magnification; scale bar = 150 *μ*m) shows extensive immune cell infiltration, pannus formation, local congestion and hemorrhage, and synovial epithelial hyperplasia. (F) Finger extension function of the right hand had recovered at the 3‐month postoperative follow‐up.

The core surgical procedure was staged tendon reconstruction:

1. Debridement of degenerated tendon stumps and inflamed synovial tissue.
2. Reconstruction of the ring finger extensor tendon: The palmaris longus tendon was harvested from the ipsilateral forearm as a free graft (approximately 4.0 cm in length) and sutured end‐to‐end to the proximal and distal stumps with 3‐0 nonabsorbable braided sutures.
3. Reconstruction of the middle finger extensor tendon: The distal stump of the middle finger extensor tendon was sutured end‐to‐side to the ulnar border of the intact extensor indicis tendon with 4‐0 nonabsorbable sutures, with tension adjusted to maintain the finger in neutral extension position.
4. Tenodesis of the extensor digiti minimi tendon: The extensor digiti minimi tendon was found to be intact but significantly lax, lacking resting tension. If left untreated, this laxity could result in weak or uncoordinated small‐finger extension, a residual extensor lag, and an imbalanced fist because the small finger would not extend synchronously with the adjacent digits. Alternative strategies were considered, including side‐to‐side tenodesis to the reconstructed extensor digitorum communis of the ring finger and simple acceptance of the laxity. However, coupling the small finger to a freshly grafted ring‐finger reconstruction was judged less reliable because the healing graft should not bear additional load, and accepting the laxity would leave a functional deficit. Tightening the extensor digiti minimi and fixing it to the dorsal wrist capsule was therefore selected as a low‐morbidity tenodesis that restores stable resting tension to the fifth extensor compartment without overloading the freshly reconstructed tendons.

Postoperatively, the right wrist and fingers were immobilized in a functional splint with the wrist in 30° extension and the metacarpophalangeal joints in 0° extension for 4 weeks. The rehabilitation protocol was staged as follows:

• Weeks 1–4: passive range of motion exercises for finger flexion within the splint to prevent peritendinous adhesion.
• Weeks 4–6: short‐arc active extension exercises, gradually increasing range of motion, with splint protection between exercises.
• Weeks 6–12: progressive strengthening exercises and functional task training, with gradual return to daily and work activities.

Pathological examination of the resected tissue showed extensive inflammatory cell infiltration, pannus formation, local congestion and hemorrhage, and synovial epithelial hyperplasia, consistent with chronic inflammatory tenosynovitis (Figure 2D,E).

### 2.4. Follow‐Up and Outcomes

The patient was discharged on postoperative Day 3 with wound healing by primary intention. Following surgery, prednisone was initiated for RA control. Further insulin adjustments were performed during external follow‐up. At the 6‐week follow‐up, the surgical incision had healed completely, and active finger extension was clinically estimated to have reached approximately 80% of the contralateral side (by visual clinical estimation rather than formal goniometric assessment). At the 3‐month follow‐up (December 20, 2025), the patient had recovered full active and passive extension of the right middle and ring fingers, with no pain at rest or during activity (Figure 2F); these findings represent favorable short‐term functional outcomes. We used the Quick Disabilities of the Arm, Shoulder and Hand (QuickDASH) score for patient‐reported functional assessment; the patient′s score decreased from 78 points preoperatively (severe upper extremity disability) to 6 points at 3 months postoperatively, representing favorable short‐term functional recovery. This 72‐point improvement exceeds the minimal clinically important difference (MCID) of 15.9 points for the QuickDASH [8], although MCID values are population‐ and condition‐dependent and are primarily intended for interpreting group‐level changes; the observed improvement is therefore reported descriptively for this individual case. Follow‐up radiographs showed no progression of wrist arthritic changes compared with baseline (Figure 1G).

Endocrinological follow‐up on October 15 showed elevated anticyclic citrullinated peptide antibody (233.49 U/mL, reference: 0–18) and rheumatoid factor (30.90 IU/mL, reference: 0–15.90), with CRP within normal range (3.22 mg/L). To assess treatment adherence, we conducted verbal follow‐up interviews at each outpatient visit and reviewed daily rehabilitation logs recorded by the patient. The patient fully complied with splint wearing rules and staged rehabilitation training schedules throughout the 12‐week recovery period; no missed exercise sessions or early splint removal were reported. No adverse events or surgical complications occurred during the 3‐month follow‐up. The patient has returned to full‐time teaching, including blackboard writing and 3–4 h of daily computer use, without pain. However, 3 months is short for tendon reconstruction; objective range of motion, extension lag, grip strength, recurrent rupture, graft elongation, adhesions, and sustained return‐to‐work require 6‐ to 12‐month or longer follow‐up and remain unavailable. The patient is being followed longitudinally, and these favorable 3‐month results should be regarded as encouraging but preliminary.

### 2.5. Patient Perspective

The patient shared her subjective experience of disease progression and treatment outcomes. Before tendon rupture, she described persistent dull aching pain over the dorsal wrist that worsened after daily typing and blackboard writing; she tried to reduce working hours but still could not relieve hand fatigue. When active finger extension was completely lost, she reported severe anxiety about losing teaching ability and being unable to perform basic self‐care such as dressing and holding tableware independently. After surgery and staged rehabilitation, she stated that splint immobilization brought mild stiffness in the early postoperative period, but the progressive short‐arc exercise program was tolerable and significantly improved daily hand function. At the 3‐month follow‐up, she expressed satisfaction with functional recovery: She could finish daily teaching work including writing on the blackboard and operating a computer for 3–4 hours without obvious pain, and her worries about career interruption were fully alleviated.

## 3. Discussion

Although the Humbert et al. classification system describes Types 1–3 MAS based on specific autoimmune disease combinations, this triad (RA, T1DM, and HT) does not conform to those established patterns [9–12]. For clarity and broader clinical applicability, we adopt the widely accepted working definition of MAS as the co‐occurrence of three or more autoimmune diseases [6, 7], without emphasizing a specific subclassification. Despite medical adjustment for rheumatological and endocrinological conditions, the patient progressed from tenosynovitis to complete tendon rupture within 2 weeks. This rapid course is notable; however, whether it reflects a more aggressive phenotype associated with multiple comorbidities or simply the natural history of inadequately controlled RA tenosynovitis with superimposed metabolic and biomechanical stressors cannot be determined from this single case.

RA is a well‐established and sufficient cause of extensor tendon rupture, and this case is consistent with RA‐driven pathogenesis. Histopathology confirmed chronic inflammatory tenosynovitis with pannus formation, and radiographs showed DRUJ involvement suggestive of mechanical attrition. Intraoperatively, the middle and ring finger extensor digitorum communis tendons were ruptured at the dorsal wrist (Zones V–VI, fourth compartment), with fibrous scar replacement of the stumps—features of chronic attritional rupture rather than acute transection. Although Vaughan‐Jackson–type attrition classically affects ulnar‐sided digits, the pattern here may reflect broader DRUJ‐related mechanical irritation combined with diffuse inflammatory tenosynovitis. Thus, RA‐related inflammatory and mechanical processes are the primary driver, with T1DM and HT as potential modifying factors whose individual contributions cannot be quantified.

The pathogenesis of tendon rupture in this case is likely multifactorial, with RA as the established primary driver and T1DM and HT as potential modifying factors. Chronic RA synovitis directly invades tendon tissue, disrupts blood supply and degrades tendon matrix [1, 2]; persistent hyperglycemia from T1DM induces abnormal collagen cross‐linking and impairs tenocyte repair capacity [13]; and HT may further disrupt collagen synthesis and tendon homeostasis [14, 15]. Each of these conditions may have contributed to tendon vulnerability in this patient, but their relative contributions and any potential synergistic interaction cannot be determined from a single case. Superimposed occupational overuse from daily keyboard work may have added cumulative biomechanical stress to the already compromised tendons, although this remains a hypothesis that cannot be established as causal from this single case. Existing literature on spontaneous tendon rupture focuses predominantly on elderly patients with isolated RA. This case extends current evidence by illustrating that early‐onset severe tendon injury can occur in a young RA patient with additional autoimmune and metabolic comorbidities, which may represent additive risk factors; the relative contribution of each condition remains undetermined. Additionally, the patient′s long‐term use of tofacitinib, a JAK inhibitor, warrants consideration. Although musculoskeletal adverse effects including tendinopathy have been reported with JAK inhibitor therapy, the histological findings in this case—extensive inflammatory cell infiltration, pannus formation, and synovial epithelial hyperplasia—are characteristic of RA‐driven inflammatory tenosynovitis rather than drug‐induced tendinopathy. Nevertheless, a potential contributory effect of tofacitinib on tendon vulnerability cannot be entirely excluded and should be monitored in future cases.

This case illustrates an instance of clinical imaging discordance. Despite complete loss of active extension of two digits, initial ultrasonography detected only tenosynovitis and a ganglion cyst and did not identify the complete tendon rupture. This may reflect the difficulty of distinguishing chronic tendon rupture with scar formation from intact but thickened tendons on ultrasound, particularly in the setting of surrounding inflammatory fluid [16, 17]. This single case does not permit a general conclusion about the sensitivity of ultrasonography for tendon rupture. Rather, it underscores that when substantial functional loss is not explained by initial imaging findings, clinicians should consider repeat expert ultrasonography, MRI, or surgical exploration to establish the diagnosis.

Clinically, this case supports the implementation of a multidisciplinary management strategy for MAS patients, involving rheumatologists, endocrinologists, and hand surgeons. Key perioperative considerations include optimization of glycemic control, careful management of immunosuppressive agents (particularly JAK inhibitors, which may affect wound healing and infection risk), and proactive treatment of intercurrent infections such as candidiasis. For patients with high‐demand hand occupations, enhanced monitoring of tendon health and targeted occupational protection guidance may be considered as reasonable clinical measures to potentially prevent irreversible tendon injury, although this case does not establish their efficacy.

## 4. Take Home Messages

In summary, this case highlights several critical take‐home messages:

1. Early‐onset spontaneous tendon rupture can occur in young RA patients with additional autoimmune and metabolic comorbidities; whether these conditions act synergistically or represent additive risk, factors cannot be determined from a single case.
2. When clinical functional loss is disproportionate to initial ultrasound findings, clinicians should maintain a low threshold for repeat expert‐performed ultrasonography or MRI; surgical exploration may be considered for definitive diagnosis when imaging remains inconclusive for tendon rupture.
3. Combined tendon reconstruction (free graft + end − to − side suture + tenodesis) is a feasible treatment strategy for multidigit extensor tendon rupture with extensive tissue defects; early functional results at 3 months are encouraging, but longer follow‐up is needed to confirm durability.
4. Multidisciplinary collaboration and occupational protection guidance may be considered for MAS patients with high hand workload, although their preventive efficacy remains to be established.

## 5. Reflections and Limitations

This case has several limitations. First, the patient received subsequent rheumatological and endocrinological management at an external hospital, so long‐term medication adherence and disease activity data are unavailable. We attempted to contact the receiving center to obtain follow‐up information; however, due to patient privacy regulations and interinstitutional data sharing restrictions, these records could not be obtained. Second, as a single case report, the findings cannot be generalized to all MAS patients. Third, the follow‐up duration of 3 months is relatively short for tendon reconstruction; the durability of the functional recovery, including potential graft elongation, adhesion formation, and recurrent rupture, remains to be determined with longer term follow‐up. For future comparable clinical scenarios, earlier MRI should be considered when ultrasound results cannot account for the severity of functional impairment. Preoperative multidisciplinary consultation to optimize glycemic control and disease activity may also improve postoperative recovery and reduce recurrence risk.

## Author Contributions

Chuancheng Huang: conceptualization, data curation, formal analysis, investigation, methodology, resources, software, writing—original draft, and writing—review and editing. Baoguo Cheng: data curation, investigation, and writing—review and editing. Deshuai Tian and Dawei Wang: resources, visualization, and writing—review and editing. Bin Wang: data curation, and writing—review and editing. Chen Xie: conceptualization, formal analysis, and writing—review and editing. Zongyu Li (corresponding author): conceptualization, funding acquisition, project administration, supervision, validation, writing—review and editing.

## Funding

This study was supported by the Natural Science Foundation of Shandong Province (10.13039/501100007129; ZR2023MC177).

## Disclosure

The authors bear full responsibility for the manuscript. The funding bodies did not participate in study design, data analysis, manuscript preparation or submission approval. All authors have read and approved the final manuscript.

## Ethics Statement

This is a retrospective case report, and all clinical management constituted standard care. Public dissemination of the complete clinical record was reviewed and approved by the Ethics Committee of the 960th Hospital of the Chinese People′s Liberation Army on December 10, 2025 (Approval No. 2025‐257). Written informed consent for publication was obtained from the patient on December 20, 2025.

## Conflicts of Interest

We declare that we have no financial or personal relationships with individuals or organizations that could improperly affect our research results. No professional or personal interests related to any commercial products, services, or enterprises exist that might bias the conclusions reported in this manuscript.

## Supporting information


**Supporting Information** Additional supporting information can be found online in the Supporting Information section. 

## Data Availability

The data supporting the findings of this case report are available from the corresponding author upon reasonable request.
