# Supplementary material for: Case Report: Spontaneous Extensor Tendon Rupture of the Middle and Ring Fingers in a Patient With Multiple Autoimmune Syndrome
Source: Case Rep Orthop. 2026 Nov 6;2026:4977682. doi: 10.1155/cro/4977682 (PMC13741286; doi:10.1155/cro/4977682)
Supplement: Supplementary file 1 [file CRO-2026-4977682-s001.docx]

# CARE Checklist Self-Assessment for the Manuscript

Manuscript title: **Case Report: Spontaneous Extensor Tendon Rupture of the Middle and Ring Fingers in a Patient with Multiple Autoimmune Syndrome**

| **Topic** | **Item No.** | **Checklist Item Description** | **Self-Assessment Result** | **Notes (Location in Manuscript)** |
| --- | --- | --- | --- | --- |
| Title | 1 | The diagnosis or intervention of primary focus followed by the words "case report" | √ | The title leads with the core clinical condition and includes "Case Report" explicitly. Location: Title Page & opening of main text |
| Key Words | 2 | 2 to 5 key words that identify diagnoses or interventions in this case report, including "case report" | √ | The current keyword list contains 3 terms (including "Case Report"). Location: below Abstract |
| Abstract (no references) | 3a | Introduction: What is unique about this case and what does it add to the scientific literature? | √ | The abstract states that spontaneous extensor tendon rupture in a young patient is uncommon, and describes unusually early-onset severe tendon rupture in a young RA patient with additional autoimmune and metabolic comorbidities, highlighting the potential need for enhanced monitoring and occupational protection. Location: Abstract, sentences 2 & 6 |
|  | 3b | Main symptoms and/or important clinical findings | √ | The abstract describes the patient’s initial presentation of dorsal hand pain/masses and subsequent limited finger extension, along with the background of three autoimmune diseases. Location: Abstract, sentences 3–4 |
|  | 3c | The main diagnoses, therapeutic interventions, and outcomes | √ | The abstract specifies the core diagnoses (RA, T1DM, hypothyroidism, tendon rupture), surgical interventions (tendon grafting, end-to-side suture, tenodesis), and 3-month functional recovery outcome. Location: Abstract, sentences 4–5 |
|  | 3d | Conclusion – What is the main "take-away" lesson(s) from this case? | √ | The abstract concludes that this case describes unusually early-onset severe tendon rupture in a young RA patient with additional autoimmune and metabolic comorbidities, highlighting the potential need for enhanced monitoring and occupational protection in patients with multiple systemic risk factors and high hand workload. Location: Abstract, final sentence |
| Introduction | 4 | One or two paragraphs summarizing why this case is unique (may include references) | √ | The Introduction section consists of two paragraphs, summarizes the current evidence gap, and explicitly states two publication-worthy merits of the case (rare clinical phenotype, complex multidisciplinary decision-making), with supporting references. Location: Introduction section |
| Patient Information | 5a | De-identified patient specific information | √ | Only age, gender and occupation are provided, with no name, ID number, address or other identifiable information. Location: Case Presentation → Patient Information subsection |
|  | 5b | Primary concerns and symptoms of the patient | √ | Reports the patient's chief complaints at initial presentation: painful swelling on the dorsum of the right hand and accompanying vulvar pruritus; complete loss of active finger extension developed 2 weeks later. Location: Case Presentation → Patient Information subsection |
|  | 5c | Medical, family, and psycho-social history including relevant genetic information | √ | Details the full medical history of three autoimmune diseases, negative family history of autoimmune diseases, and occupational context of long-term keyboard use. Location: Case Presentation → Patient Information subsection |
|  | 5d | Relevant past interventions with their outcomes | √ | Lists the patient’s long-term medication regimen for immunosuppression and hormone replacement therapy prior to admission. Location: Case Presentation → Patient Information subsection |
| Clinical Findings | 6 | Describe significant physical examination (PE) and important clinical findings | √ | Documents physical examination findings (tender mass, 0/5 extensor strength, loss of active extension), along with key abnormal laboratory results. Location: Case Presentation → Diagnostic Assessment subsection |
| Timeline | 7 | Historical and current information from this episode of care organized as a timeline | √ | The clinical course is presented in strict chronological order: 5 Sep initial visit → 8 Sep laboratory assessment → 23 Sep emergency surgery → 15 Oct endocrinology follow-up → 20 Nov 3-month surgical follow-up. Location: entire Case Presentation section |
| Diagnostic Assessment | 8a | Diagnostic testing (such as PE, laboratory testing, imaging, surveys) | √ | Covers physical examination, serological tests, plain radiography, MRI, ultrasonography and histopathological examination. Location: Case Presentation → Diagnostic Assessment subsection |
|  | 8b | Diagnostic challenges (such as access to testing, financial, or cultural) | √ | Identifies the key diagnostic limitation: initial ultrasonography failed to detect complete tendon rupture, leading to delayed definitive diagnosis. Location: Discussion section, second paragraph |
|  | 8c | Diagnosis (including other diagnoses considered) | √ | Provides 4 definitive diagnoses with ICD-10 codes; initial differential considerations included tenosynovitis and ganglion cyst. Location: Case Presentation → Diagnostic Assessment subsection |
|  | 8d | Prognosis (such as staging in oncology) where applicable | Not applicable | This is a non-oncological musculoskeletal case; prognostic staging is not clinically indicated. |
| Therapeutic Intervention | 9a | Types of therapeutic intervention (such as pharmacologic, surgical, preventive, self-care) | √ | Includes three categories of intervention: pharmacologic (medication adjustment), surgical (tendon reconstruction), and rehabilitative (postoperative staged rehabilitation protocol). Location: Case Presentation → Therapeutic Intervention subsection |
|  | 9b | Administration of therapeutic intervention (such as dosage, strength, duration) | √ | Specifies exact drug dosages, detailed surgical steps (graft source, suture method, tension adjustment), and staged rehabilitation timeline with exercise content. Location: Case Presentation → Therapeutic Intervention subsection |
|  | 9c | Changes in therapeutic intervention (with rationale) | √ | Documents perioperative medication adjustments: preoperatively, tofacitinib was withheld and insulin dosage was adjusted for glycemic control (target 7.8–10.0 mmol/L); postoperatively, prednisone was initiated for RA control, with further insulin adjustments performed during external follow-up. Location: Case Presentation, second paragraph of clinical course |
| Follow-up and Outcomes | 10a | Clinician- and patient-assessed outcomes (if available) | √ | Clinician evaluation: wound healing, joint range of motion, imaging; Patient-reported: QuickDASH disability score before and after surgery. *Location: Case Presentation → Follow-up and Outcomes* |
|  | 10b | Important follow-up diagnostic and other test results | √ | Reports follow-up laboratory markers (anti-CCP, RF, CRP) and radiographic findings at 1 month postoperatively. Location: Case Presentation → Follow-up and Outcomes subsection |
|  | 10c | Intervention adherence and tolerability (How was this assessed?) | √ | Adherence assessed via outpatient verbal interviews and patient self-recorded rehabilitation logs; all interventions well tolerated. *Location: Case Presentation → Follow-up and Outcomes* |
|  | 10d | Adverse and unanticipated events | √ | Explicitly states that no adverse events or surgical complications occurred during the follow-up period. Location: Case Presentation → Follow-up and Outcomes subsection |
| Discussion | 11a | A scientific discussion of the strengths AND limitations associated with this case report | √ | The Reflections and Limitations section clearly states the limitations (loss to external follow-up, single-case design); the strengths (rare MAS phenotype, multidisciplinary management insight) are addressed in the main discussion. Location: Reflections and Limitations section |
|  | 11b | Discussion of the relevant medical literature with references | √ | The discussion cites 15 references to contextualize the pathogenesis, diagnostic challenges and treatment principles, and compares this case with existing evidence. Location: Discussion section |
|  | 11c | The scientific rationale for any conclusions (including assessment of possible causes) | √ | Discusses potential multifactorial contributors to tendon rupture (RA synovitis as the primary and sufficient driver, with diabetic collagen damage, hypothyroid metabolic disturbance, tofacitinib use, and occupational overuse as potential modifying factors), while explicitly acknowledging that the relative contribution of each comorbidity and any synergistic interaction cannot be determined from this single case. Location: Discussion section, first paragraph |
|  | 11d | The primary "take-away" lessons of this case report (without references) in a one-paragraph conclusion | √ | A standalone Take Home Messages section summarizes 4 core clinical lessons without citations. Location: Take Home Messages section |
| Patient Perspective | 12 | The patient should share their perspective in one to two paragraphs on the treatment(s) they received | √ | The dedicated subsection recording patient’s preoperative anxiety, postoperative rehabilitation experience and long-term functional satisfaction. *Location: Case Presentation → Patient Perspective subsection* |
| Informed Consent | 13 | Did the patient give informed consent? Please provide if requested. | √ | The Ethics statement explicitly confirms that written informed consent was obtained from the patient for publication. Location: Statements & Declarations → Ethics statement |
